# Evaluation of the anti-inflammatory, antioxidant, and cytotoxic potential of *Cardamine amara* L. (Brassicaceae): A comprehensive biochemical, toxicological, and *in silico* computational study

**DOI:** 10.3389/fchem.2022.1077581

**Published:** 2023-01-06

**Authors:** Abdul Basit, Saeed Ahmad, Kashif ur Rehman Khan, Hanan Y. Aati, Asmaa E. Sherif, Chitchamai Ovatlarnporn, Safiullah Khan, Huma Rao, Muhammad Adeel Arshad, Muhammad Nadeem Shahzad, Shagufta Perveen

**Affiliations:** ^1^ Department of Pharmaceutical Chemistry, Faculty of Pharmaceutical Sciences, Prince of Songkla University, Songkhla, Thailand; ^2^ Drug Delivery System Excellence Center, Prince of Songkla University, Songkhla, Thailand; ^3^ Department of Pharmaceutical Chemistry, Faculty of Pharmacy, The Islamia University of Bahawalpur, Bahawalpur, Pakistan; ^4^ Department of Pharmacognosy, College of Pharmacy, King Saud University, Riyadh, Saudi Arabia; ^5^ Department of Pharmacognosy, College of Pharmacy, Prince Sattam Bin Abdul Aziz University, Alkharj, Saudi Arabia; ^6^ Department of Pharmacognosy, Faculty of Pharmacy, Mansoura University, Mansoura, Egypt; ^7^ Department of Pharmaceutics, Faculty of Pharmacy, The Islamia University of Bahawalpur, Bahawalpur, Pakistan; ^8^ Department of Pharmaceutical Chemistry, Institute of Pharmacy, Faculty of Pharmaceutical and Allied Health Sciences, Lahore College for Women University, Lahore, Pakistan; ^9^ Department of Chemistry, School of Computer, Mathematical and Natural Sciences, Morgan State University, Baltimore, MD, United States

**Keywords:** *Cardamine amara*, UPLC-Q-TOF-MS, cytotoxicity, anti-inflammatory, oxidative stress, molecular docking

## Abstract

**Introduction:** *Cardamine amara* L. (Brassicaceae) is an important edible plant with ethnomedicinal significance. This study aimed at evaluating the phytochemical composition, anti-inflammatory, antioxidant and cytotoxicity aspects of the hydro-alcoholic extract of *C. amara* (HAECA).

**Methods:** The phytochemical composition was evaluated through total phenolic contents (TPC), total flavonoid contents (TFC) determination and UPLC-QTOF-MS profiling. Anti-inflammatory evaluation of HAECA was carried out through the carrageenan induced paw edema model. Four *in vitro* methods were applied in the antioxidant evaluation of HAECA. MTT assay was used to investigate the toxicity profile of the species against human normal liver cells (HL7702), human liver cancer cell lines (HepG2) and human breast cancer cell lines (MCF-7). Three major compounds (Gentisic acid, skullcapflavone and conidendrine) identified in UPLC-Q-TOF-MS analysis were selected for *in silico* study against cyclooxygenase (COX-I and COX-II).

**Results and Discussion:** The findings revealed that HAECA is rich in TPC (39.32 ± 2.3 mg GAE/g DE) and TFC (17.26 ± 0.8 mg RE/g DE). A total of 21 secondary metabolites were tentatively identified in UPLC-Q-TOF-MS analysis. In the MTT cytotoxicity assay, the extract showed low toxicity against normal cell lines, while significant anticancer activity was observed against human liver and breast cancer cells. The carrageenan induced inflammation was inhibited by HAECA in a dose dependent manner and showed a marked alleviation in the levels of oxidative stress (catalase, SOD, GSH) and inflammatory markers (TNF-α, IL-1β). Similarly, HAECA showed maximum antioxidant activity through the Cupric reducing power antioxidant capacity (CUPRAC) assay (31.21 ± 0.3 mg TE/g DE). The *in silico* study revealed a significant molecular docking score of the three studied compounds against COX-I and COX-I. Conclusively the current study encourages the use of *C. amara* as a novel polyphenolic rich source with anti-inflammatory and antioxidant potential and warrants further investigations on its toxicity profile.

## 1 Introduction

The inflammatory process has become a potential target for treating and managing various disorders, such as cancer, emphysema, diabetes, and, more recently, coronavirus disease 2019 (COVID-19). Therefore, methods to ameliorate this condition have attracted attention. The underlying factors contributing to inflammation include oxidative stress (Yesmin et al., 2020), which can be described as an imbalance between the antioxidant defense mechanism and free radicals such as superoxide anion, hydroxyl, hydroperoxyl, and peroxyl (Jones, 2006). Oxidative stress, in turn, leads to the overexpression of inflammatory mediators such as TNF-α and IL-1β. These markers then initiate and maintain inflammation (Ansari et al., 2020). Many studies have demonstrated the involvement of oxidative stress-mediated inflammation in the etiology of many diseases such as diabetes, aging, skin tissue damage, and peptic ulcers (Tandon et al., 2004; Babu et al., 2015; Asmat et al., 2016). Therefore, drug candidates with ameliorative effects on oxidative stress are being considered suitable alternatives for treating various disorders (Pieczykolan et al., 2021). The approach of investigating medicinal plants for therapeutic agents has proven to be a useful and productive tool in recent decades. We examined *Cardamine amara* L. for its anti-inflammatory potential through the carrageenan-induced paw edema model and possible anti-inflammatory mechanisms by estimating the levels of oxidative stress and inflammatory markers. *C. amara* belongs to the family Brassicaceae, also known as Cruciferae. The family comprises many edible species that are used as oil seeds, food, condiments, and vegetables. The family contains important genera with impactful species in medicinal, agronomical, and economical contexts (Raza et al., 2020). *Cardamine* is one of the largest genera of Brassicaceae and contains at least 200 species (Lihová et al., 2006). Various *Cardamine* species have been investigated phytochemically and pharmacologically (Montaut and Bleeker, 2013). Still, several species of the genus have not been explored yet. Among these, *C. amara* is locally known as zerucha or large bittercress with synonyms *Cardamine triphylla* Pall, *Cardamine nasturtiana* Thuill.*,* and *Cardamine umbrosa* Lej. (www.theplantlist.com) (Luczaj, 2012). This perennial plant grows up to 0.6 m, flowers from April to June, and seeds from May to September. The species has a wide distribution extending from Europe to Asia (Lihová et al., 2000). This non-conventional food plant has been used as a food additive in meat dishes and soups in Slovakia and Estonia and ethnomedicinally used as an ascourbatic, diuretic, and stimulant. To our knowledge and literature search, limited literature has reported on the pharmacological and phytochemical aspects of this species. The toxicity concerns and ethnomedicinal importance of *C. amara* led us to design the present study on the phytochemical profile, cytotoxicity, and anti-inflammatory and antioxidant activities of *C. amara.* The application of sophisticated spectroscopic techniques for the identification of chemical constituents in the extracts of natural sources has increased markedly. Various instruments such as ultra-performance liquid chromatography (UPLC) coupled with quadrupole time-of-flight mass spectrometry (Q-TOF-MS/MS) make the process of identification convenient and efficient (Qi et al., 2014). Therefore, the current study applied UPLC-Q-TOF-MS technique to comprehensively evaluate the phytochemical composition of *C. amara.*

## 2 Materials and methods

### 2.1 Collection of plant material and preparation of extract

Whole-plant material was obtained from Bannu District, Khyber Pakhtunkhwa, Pakistan, in April 2019. The collected plant was identified by Mr. Ghulam Sarwar, Taxonomist at the Department of Life Sciences, The Islamia University of Bahawalpur (IUB), Punjab, Pakistan, and voucher number 789/LS was deposited at the herbarium of the same University. The plant material was washed and shade-dried, followed by grinding into a fine powder. The powdered material (1 kg) was macerated with hydroalcoholic solvent (methanol 80%: water 20%) for 15 days with occasional stirring and then filtered through Whatman No.1 filter paper. The filtrate was then concentrated on a rotary evaporator at 40°C. The percent yield of the extract was determined on a dry weight basis according to the following formula:

Percent yield: weight of the dry extract/weight of the dry plant material * 100.

### 2.2 Phytochemical composition

#### 2.2.1 Determination of the total bioactive content

The total bioactive content in hydro-alcoholic extract of *C. amara* (HAECA) was evaluated by determining the total phenolic content (TPC) and total flavonoid content (TFC). TPC and TFC were determined using the Folin–Ciocalteu and aluminum trichloride methods, respectively. The standard protocol (Uysal et al., 2018) was used. The TPC readings were recorded as milligrams of gallic acid equivalents per gram of dry extract (mg GAE/g DE), while TFC was recorded as milligrams of rutin equivalents per gram of dry extract (mg RE/g DE).

#### 2.2.2 Phytochemical profiling using UPLC-Q-TOF-MS analysis

The extract was subjected to UPLC-Q-TOF-MS analysis for the identification of phytochemicals. This analysis applied Triple TOF™ 5600 (AB Sciex, Foster, USA) LC-MS mass spectrometry with a DuoSpray TM ion source for the phytochemical profiling of HAECA using previously established protocols (Basit et al., 2022a). Chromatographic separation was performed using a Prominence. The HR-MS/MS Spectral Library 1.0 Software Database, previous literature, and the METLIN database were used to identify the phytoconstituents (Hu et al., 2020).

### 2.3 *In vitro* cytotoxicity assay

#### 2.3.1 Cell culture

This study used three cell lines: human breast cancer cells (MCF-7), human liver cancer cells (HepG2), and normal human liver cells (HL7702). The cell lines were obtained from the Cell Bank of the University of Veterinary and Animal Sciences, Lahore, Punjab, Pakistan, and grown in Roswell Park Memorial Institute Medium (RPMI) and modified Eagle’s medium (DMEM) in a gas incubator at 37°C, 5% CO_2_, and 95% humidity. The cell cultures were supplemented with non-essential amino acids (1%), PEST (1%), and 10% fetal bovine serum.

#### 2.3.2 Evaluation of cytotoxicity using MTT assay

The cytotoxicity of HAECA in human breast cancer cells (MCF-7), human liver cancer cells (HepG2), and normal human liver cells (HL 7702) was evaluated by (3-(4,5-dimethylthiazol-2-yl)-2,5-diphenyltetrazolium bromide) tetrazolium (MTT) assay as previously described (Chavan et al., 2020). The cell lines were placed in a 96-well plate with a density of 1 × 10^4^ cells per well and incubated for 24 h. HAECA (300, 200, 100, 50, and 25 μg/ml) was then added at 24 h and 48 h. MTT reagent was added at a concentration of 5 mg/ml in 20 ml of phosphate buffer saline per well and incubated for 4 h with CO_2_ followed by the removal of medium and addition of DMSO (150 μL) for the dissolution of formazan. Absorbance was measured at 490 nm using a microplate reader (BioTek, USA) for cell viability assessment. The results were presented as % cell viability and the IC_50_ was calculated for HepG2 and MCF-7 cells.

### 2.4 *In vitro* antioxidant potential

The antioxidant potential of HAECA was evaluated using *in vitro* methods, namely, 2,2 diphenyl-1-picrylhydrazyl (DPPH), 2,2 azinobis (3-ethylbenothiazoline)-6-sulfonic acid (ABTS), ferric reducing antioxidant power (FRAP), and cupric reducing antioxidant capacity (CUPRAC). The experiments were performed as we previously described (Basit et al., 2021). The antioxidant potential was expressed in terms of milligram Trolox equivalent per gram of dry extract (mg TE/g DE).

### 2.5 Evaluation of the anti-inflammatory effects of HAECA

#### 2.5.1 Animals

BALB/c mice (25–30 g) were obtained from the National Institute of Health, Rawalpindi, Punjab, Pakistan, and kept in the animal house of the Faculty of Pharmacy, The Islamia University of Bahawalpur under standard conditions with *ad libitum* access to food and water. Six mice were housed per cage. The experiment was approved by the Pharmacy Animal Ethical Committee of The Islamia University of Bahawalpur with permit number 47-2021/PAEC (Basit et al., 2021).

#### 2.5.2 Carrageenan-induced paw edema model

The anti-inflammatory effect of HAECA was assessed using the carrageenan-induced paw edema model (Yang L et al., 2020). The experimental animals were grouped into five categories (n = 6 each):

Control: Normal saline 10 ml/kg orally.

Positive control: Dexamethasone 75 mg/kg subcutaneously.

Treated groups 3–5: 100, 200, and 400 mg/kg of extract, respectively.

The irritant, 100 µL of 1% carrageenan, was administered subcutaneously to the right hind paw 1 h after treatment administration. Before carrageenan administration, the paw volume was measured and marked as the initial volume (V1), while volume (V2) was measured at 0.5, 1, 2, 4, and 6 h after carrageenan administration. The changes in mouse paw size and volume were used as an indicator for the induction of inflammation. A digimatic caliper (Mitutoyo, Japan) was used to measure the paw volumes. The following formula was used to calculate the percent inhibition of edema:

$$\%Inhibition=\frac{\left(V_{control}-V_{\;treated}\right)}{V_{\;control}\times 100}.$$

#### 2.5.3 Preparation of tissue homogenates

The experimental animals were euthanized using ketamine at a dose of 350 mg/kg at the end of the study and a portion of the paw tissue was immediately separated. Homogenates of the paw tissues (10% w/v) in 0.1 M Tris-HCl buffer of 7.4 pH were then prepared. The homogenates were sterilized and stored for the quantification of inflammatory and oxidative stress markers (Khan et al., 2020).

#### 2.5.4 Estimation of catalase, SOD, and GSH levels

The levels of catalase in the homogenates of mouse paw tissue were quantified as described previously with minor changes (Doherty et al., 2010). In this method, 3 ml of H_2_O_2_ phosphate buffer was added to 40 µL of enzyme extract in an experimental cuvette and mixed. Absorption was measured at 240 nm on a spectrophotometer. The findings were recorded as U/mg protein. Similarly, SOD levels were estimated according to the standard method with little modification (Kakkar et al., 1984). In this procedure, the reduction of nitro blue tetrazolium (NBT) to formazan mediated by superoxide anion was measured at 540 nm. The reaction was stopped when SOD was added to the mixture. The inhibition was measured as the enzyme activity and presented as U/MG protein. The GSH levels were estimated as previously described (Farombi et al., 2007) with minor changes. In this method, 0.1 ml of homogenate was added to phosphate buffer (2.5 ml) followed by the addition of DTNB (5, 5′-dithiobis-(2-nitrobenzoic acid) to a final volume of 3 ml. The absorbance was recorded at 412 nm and the readings were expressed in mmol/mg protein.

#### 2.5.5 Quantification of inflammatory markers (using ELISA kit)

Mouse paw tissue samples for the quantification of inflammatory markers were prepared as previously described (Khan et al., 2019) with minor changes. In brief, the proteins in the tissue were extracted from 100 mg of tissue. Next, 0.05% Tween-20, 0.4-M NaCl, and protease inhibitors were added to the mixture. The sample was homogenized at 3000 g for 10 min. The supernatant then collected was stored at −80°C. Commercially available kits (eBioscience, Inc, San Diego, CA) were used for the estimation of IL-1β and TNF-α levels.

### 2.6 Bioinformatic analysis

Molecular docking is an important tool for computer-aided drug design in the development of novel ligands with optimal medicinal properties. A focused database search for Protein Data Bank (PDB) format and tools for the preparation of the ligands in PDB format is needed for molecular retrieval. For this purpose, various techniques were used using different tools such as AutoDock Vina, MGLTools, PyRx, Discovery Studio, and Babel. The structures of the cyclooxygenase-I, cyclooxygenase-II, catalase, GSH, SOD, HO1, and iNOS enzymes were downloaded from PDB. The Discovery Studio 2021 client was used for further preparation of the enzyme. Three ligands (conidendrine, skullcapeflavone, and gentisic acid) were used. These compounds were selected based on the quantification results in the HPLC-PDA analysis. The structures of these ligands were downloaded from the PubChem database in structured data format (SDF). The ligands were further prepared with Babel. The prepared receptors and ligands were then uploaded to Vina, which was embedded in PyRx. These structures were placed in the active pockets using AutoDock Vina. The interactions were evaluated using Discovery Studio Visualizer (Basit et al., 2022b; Dilshad et al., 2022).

### 2.7 Statistical analysis

All assays were conducted in triplicate. The values were expressed as means ± standard deviation (*n* = 3). One-way ANOVA followed by Tukey’s test was applied using GraphPad Prism 7.0 for the analysis of the data. The different levels of comparison for the determination of significance were *p < 0.05, **p < 0.01, and ***p < 0.001.

## 3 Results and discussion

This study is the first to examine the phytochemical, toxicological, pharmacological, and biological aspects of *C. amara*. A mechanism-based approach was applied to assess the anti-inflammatory potential of HAECA. Secondary metabolites of plant origin, particularly flavonoids and phenols, have diverse pharmacological potential. Previous studies described the determination, extraction, and pharmacological effects of secondary metabolites (Ahmad et al., 2019; Shahzad et al., 2022). The selection of solvent is very important in the preparation of plant extract. The current study used a hydro-alcoholic solvent with a composition of 80% methanol and 20% distilled water in the extraction process. The rationale behind the use of this solvent system was to obtain a high yield with maximum extraction of secondary metabolites of different polarities due to the expanded polarity index of the solvent system (Zayede et al., 2020). In this study, 67 g of extract was obtained, with a 6.7% yield.

### 3.1 Phytochemical composition

**FIGURE 1:**
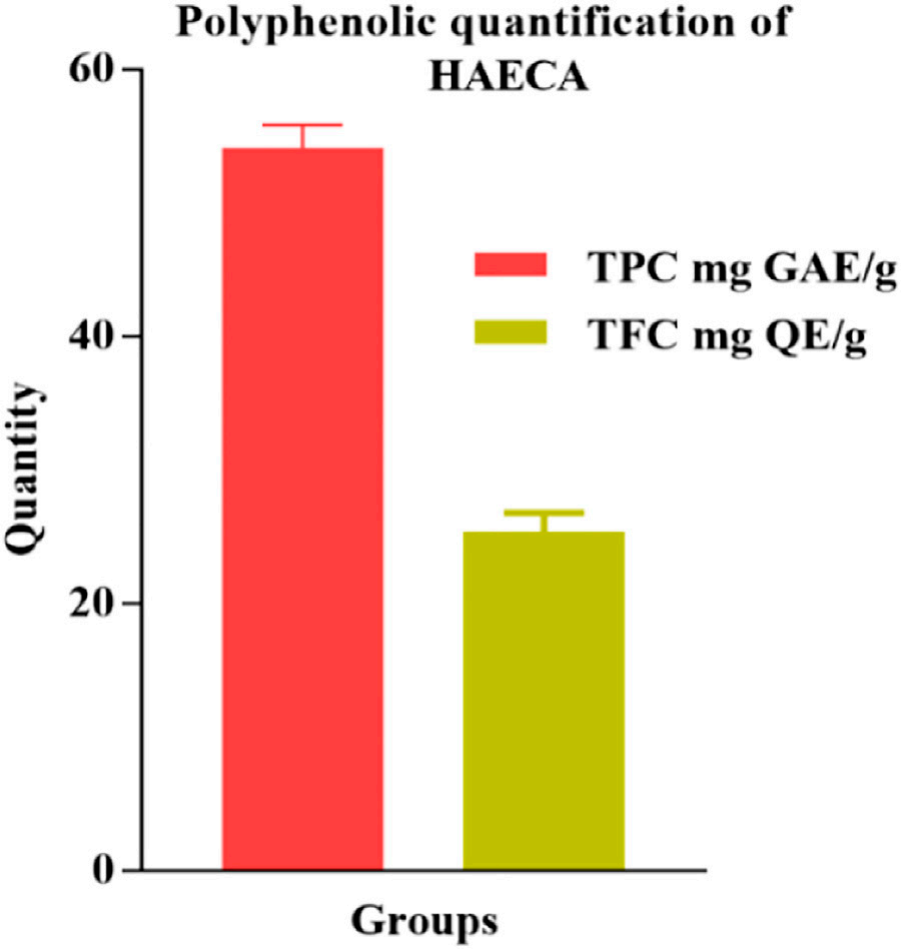
Total polyphenolic content in hydro-alcoholic extract of *Cardamine amara* (HAECA).

The phytochemical profiling of HAECA was carried out through quantification of polyphenolic compounds and UPLC-Q-TOF-MS analysis. The phytoconstituents, particularly flavonoids and phenols, have attracted interest due to their antioxidant and anti-inflammatory potential (Zengin et al., 2018). In the current investigation, the total phenolic and flavonoid contents of the extract were determined. The results are displayed in Figure 1. The extract was rich in phenols (54.14 ± 2.3 mg GAE/g DE) and flavonoids (25.39 ± 0.8 mg RE/g DE). The polyphenolic compounds are proved with pharmacological significance in various previous reports (Ahmad et al., 2022).

**FIGURE 2:**
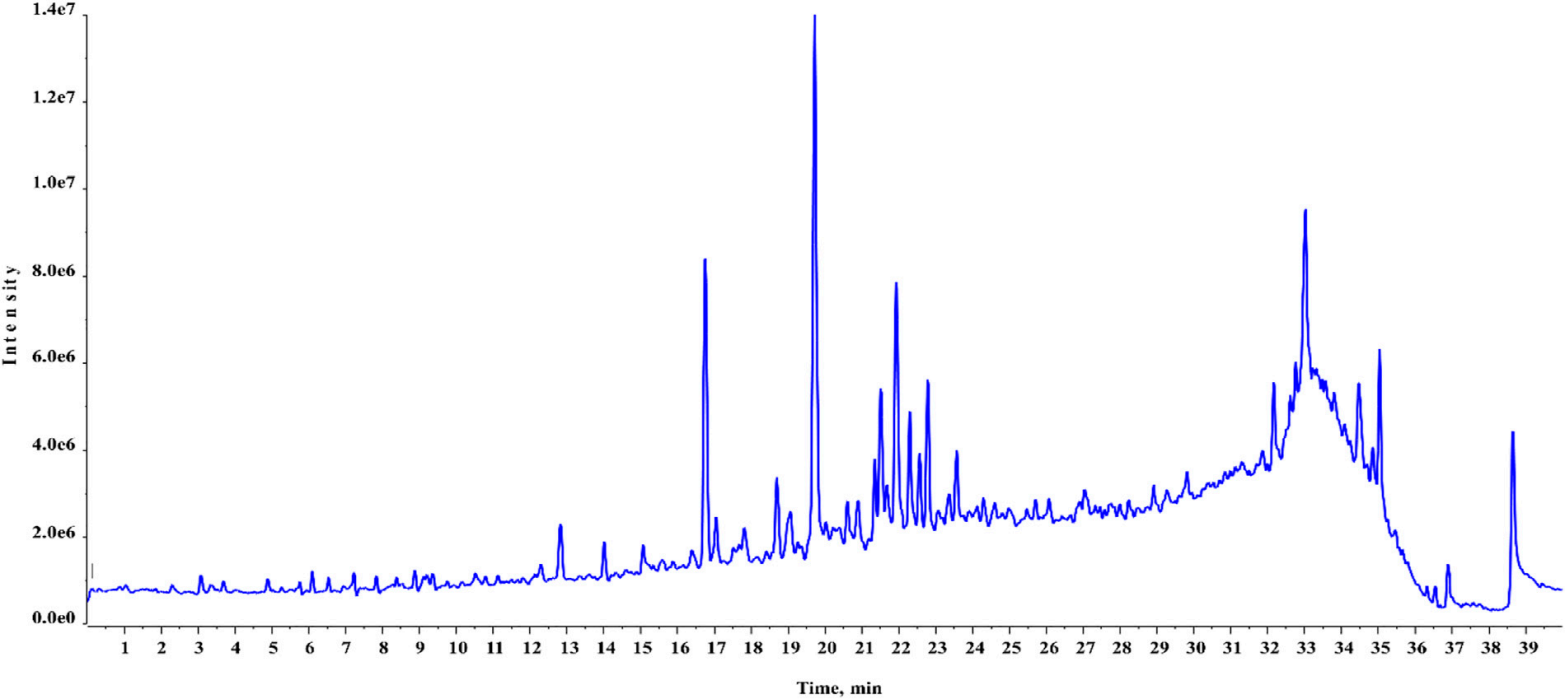
Total ionic chromatogram (TIC) of HAECA (positive mode in UPLC-Q-TOF-MS analysis).

**FIGURE 3:**
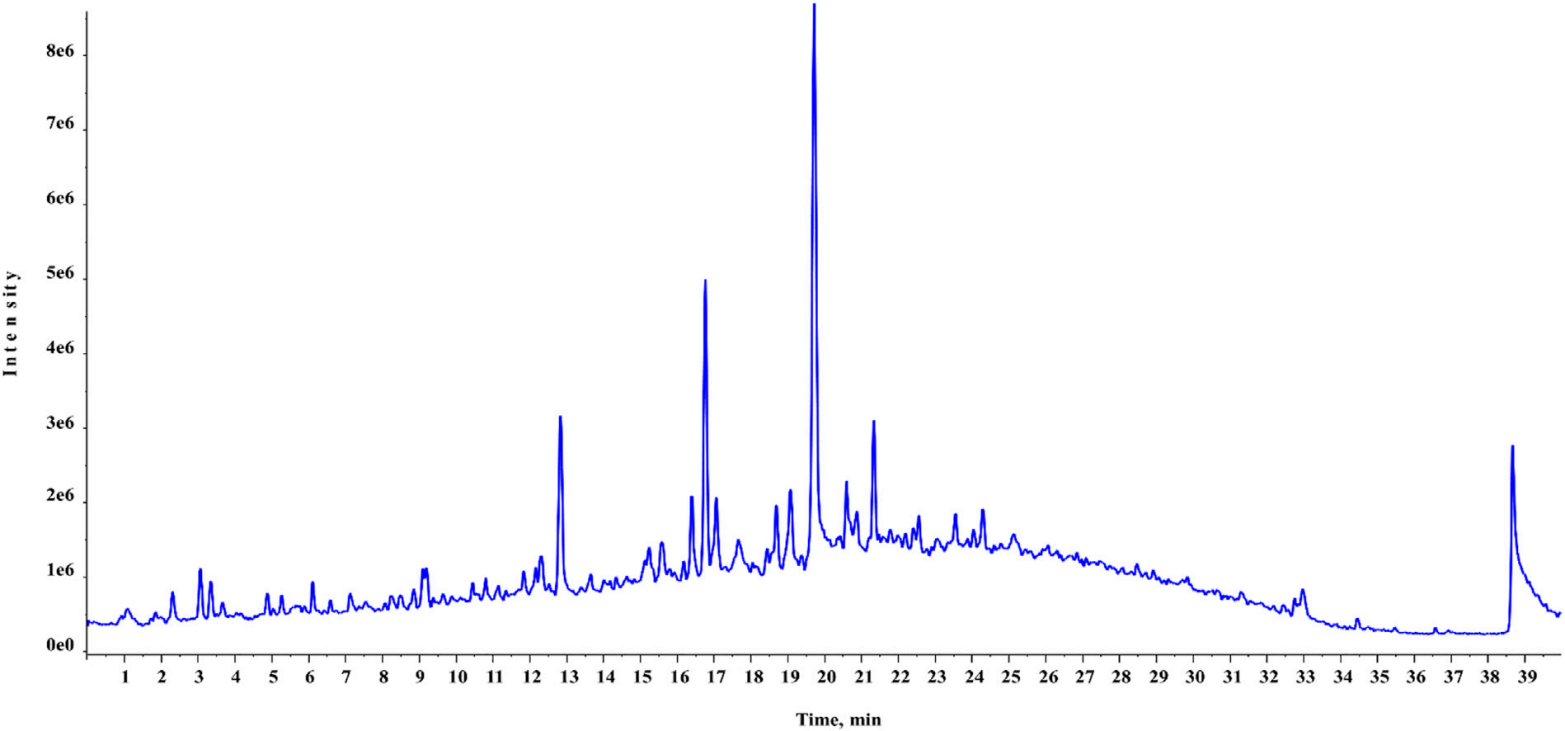
Total ionic chromatogram (TIC) of HAECA (negative mode in UPLC-Q-TOF-MS analysis).

Chemical profiling of HAECA by UPLC-Q-TOF-MS analysis revealed 21 compounds with 13 secondary metabolites in the negative mode of ionization (Table 1) and eight secondary metabolites in the positive mode of ionization (Table 2). Polyphenols were the major class of compounds. The total ionic chromatograms of the analysis are displayed in Figures 2, 3. In detail, compounds 2 and 3 showed protonated [M + H]^+^ molecules at *m/z* 199.05 and 209.08, respectively, and were tentatively identified as syringic acid and sinapaldehyde. Syringic acid showed fragments at *m/z* 162.90 [M + H-2H_2_O], a diagnostic feature of phenols (Yang B et al., 2020). Compound 8 was assigned a molecular formula of C_29_H_24_O_12_ and tentatively identified as theaflavin. Similarly, a phenolic acid cinnamoyl glucose 9 was found at *m/z* 309.09. Compound 1 showed peaks at *m/z* 127.03 and had a molecular formula of C_6_H_6_O_3_; thus, it was identified as pyrogallol, consistent with previous reports (Cunha et al., 2017; Balkrishna et al., 2021; Brito et al., 2021; Fattahi et al., 2021). This analysis tentatively identified a total of five flavonoids. The MS product ions of the compounds are widely used for the structural characterization of flavonoids for the flavonoid glycosides and flavonoids aglycon. Flavonoid glycosides show diagnostic MS product ions at CH_3_, H_2_O, CO, and CO_2_ as well as the loss of glucuronic acid (176 Da), 132 Da due to apioside residues, and 162 Da due to hexoside residues. In detail, compound 16 showed a deprotonated molecule at *m/z* 373.09, with an MS fragment pattern similar to that reported by (Hu et al., 2020) and was tentatively identified as skullcapflavone II. Similarly, compound 4 showed a protonated ion at *m/z* 257.08 and major M/S product ions at *m/z* 152.06, 211.07, and 220.09 which led to its tentative identification as flavonoid liquiritigenin with support from previous literature (Xu et al., 2013). Compound 5 showed a protonated ion at *m/z* 314.07 and was identified as cirsimaritin. Similarly, compound 10 was identified as the flavonoid dihydrooroxylin in negative mode analysis of CEJv at *m/z* 285.07. Peak 11 showed a deprotonated molecule at *m/z* 517.098 with fragment ions at 471.111 and 424.780, indicating a loss of 2C_2_H_6_O, which suggested that the compound was 6″-O-malonylgenistin (Qi et al., 2014; Chanda et al., 2019). Lignans usually show characteristic product ions in their MS spectrum due to the neutral loss of H_2_O (18 Da), hydroxyl (OH) ions, methyl radical (CH_3_*, 15.023 Da), formaldehyde (H_2_C = O, 30.010), water and formaldehyde (48.021 Da), C=O=C (43.989 Da), CH_2_CHOH (44.026 Da), guaiacylglycerol (196.0736), syringoylglycerol (226.0841 Da), or the loss of glucose moieties depending on the type of lignan molecule (Hanhineva et al., 2012; Li et al., 2014). The current analysis tentatively identified three lignans. Compounds 6, 14, and 15 showed peaks at *m/z* 417.15, 375.14, and 355.11 and were identified as 1-acetoxypinoresinol, todolactol A, and conidendrin, respectively. Peak 18 was identified as chuanfumine (Qi et al., 2014). Compound 20 showed a protonated molecule at *m/z* 547.35 with a product ion in the MS spectrum at *m/z* 549.359; thus, it was identified as an alkaloid manzamine J with molecular formula C_36_H_44_N_4_O (Ma’arif et al., 2018). A single triterpene acid corosolic acid was observed at *m/z* 473.36 and major M/S product ions at *m/z* 95.08, 203.17, 427.35, and 473.33. The data were consistent with previous literature (Duan et al., 2021). Furthermore, single coumarin was identified at *m/z* 245.09 (peak no. 17). The MS product ions displayed by the compounds were consistent with those of compounds previously reported from other species. Therefore, the compound was tentatively named marmesin. Our data were supported by previous literature (Zhuang et al., 2018; Chanda et al., 2019; Duan et al., 2021). Two fatty acids, 3-hydroxysebacic acid and methylnonadecan-1-oate, were present in HAECA. Similarly, compounds 12 and 13 showed deprotonated molecules at *m/z* 223.06 and 623.19 and were identified as hydroxymethoxycinnamic acid and verbascoside, respectively (Elshamy et al., 2020; Yang B et al., 2020).

**TABLE 1:** Tentatively identified compounds in UPLC-Q-TOF-MS (positive mode) analysis of HAECA.

Peak no.	r.t. (min)	Mol. mass	Base peak (*m/z*) [M + H]^+^	Mol. formula	Error ppm	Compound	Class	MS/MS fragment
1	3.77	126.04	127.03	C_6_H_6_O_3_	−1.2	Pyrogallol	Phenol	53.042, 69.936, 81.035, 85.969, 90.903, 109.031, 126.958, 127.037
2	6.11	198.04	199.05	C_9_H_10_O_5_	−0.7	Syringic acid	Phenol	55.019, 69.071, 79.055, 97.029, 118.920, 125.023, 136.929, 140.047, 162.902, 181.050
3	10.50	208.76	209.08	C_11_H_12_O_4_	0.5	Sinapaldehyde	Phenol	67.067, 78.046, 91.056, 106.043, 121.065, 132.903, 149.907, 167.917, 177.913, 190.937, 209.159
4	12.78	256.13	257.08	C_15_H_12_O_4_	1.2	Liquiritigenin	Flavonoid	152.062, 153.085, 211.074, 220.928, 257.197
5	14.57	314.07	315.04	C_17_H_14_O_6_	1.9	Cirsimaritin	Flavonoid	183.006, 198.908, 220900, 294.889, 313.205
6	18.76	416.18	417.15	C_22_H_24_O_8_	0.7	1-Acetoxypinoresinol	Lignan	135.044, 181.086, 353.140
7	23.64	472.25	473.36	C_30_H_48_O_4_	−1.7	Corosolic acid	Triterpene acid	95.086, 187.146, 189.165, 203.179, 205.160, 207.173, 391.352, 427.357, 409.346, 437.340, 473.337
8	29.07	566.43	565.13	C_29_H_24_O_12_	0.9	Theaflavin	Polyphenol	136.049, 225.089, 313.103, 382.157, 403.079, 565.350

r.t, retention time; error ppm, ±5.

**TABLE 2:** Tentatively identified compounds in UPLC-Q-TOF-MS (negative mode) analysis of HAECA.

Peak no.	r.t. (min)	Mol. mass	Base peak (*m/z*) [M-H]^-^	Mol. formula	Error ppm	Compound	Class	MS product ion
9	1.05	310.07	309.09	C_15_H_18_O_7_	1.7	Cinnamoyl hexose	Phenol	98.003, 131.090, 146.074, 161.096, 176.048, 191.072, 206.095, 220.070, 244.911, 265.104, 279.086, 290.854, 309.098
10	2.43	286.14	285.07	C_16_H_14_O_5_	−1.9	Dihydrooroxylin a	Flavonoid	141.038, 169.030, 171.044, 183.051, 197.023, 211.039, 227.035, 239.030, 254.023, 255.031, 269.046, 270.049
11	3.08	518.11	517.09	C_24_H_22_O_13_	0.3	6″-O-Malonylgenistin	Flavonoid	225.058, 269.081, 313.068, 357.060, 383.071, 401.088, 424.780, 471.111, 517.091
12	6.12	2245.09	223.06	C_11_H_12_O_5_	1.7	Hydroxymethoxycinnamic acid	Phenylpropanoid	78.961, 93.038, 121.029, 149.023, 164.048, 165.017, 193.015, 204.841, 205.829, 208.037
13	9.07	624.16	623.19	C_29_H_36_O_15_	2.8	Verbascoside	Phenyl ethanoid	96.973, 161.023, 179.061, 461.159, 623.090
14	12.93	376.18	375.14	C_20_H_24_O_7_	1.6	Todolactol A	Lignan	109.035, 150.034, 165.058, 191.070, 267.066, 279.065, 312.101, 327.123
15	15.31	356.09	355.11	C_20_H_20_O_6_	0.3	Conidendrine	Lignan	121.030, 122.039, 129.075, 159.079, 189.090, 219.062, 233.082, 290.892, 300.850, 340.098, 355.114
16	16.42	374.08	373.09	C_19_H_18_O_8_	3.7	Skullcapflavone II	Flavonoid	107.019, 125.027, 205.051, 300.860, 343.108, 373.156
17	17.19	246.11	245.09	C_14_H_14_O_4_	4.3	Marmesin	Coumarin	65.052, 115.066, 117.067, 131.083, 157.058, 188.081, 191.102, 219.083, 247.070
18	18.76	393.23	392.25	C_22_H_35_NO_5_	2.8	Chuanfumine	Alkaloid	274.194, 330.189, 350.220, 394.149
19		218.21	217.10	C_10_H_18_O_5_	1.4	3-Hydroxysebacic acid	Fatty acid	78.961, 96.988, 155.11, 171.105, 217.105
20	21.38	548.41	547.35	C_36_H_44_N_4_O	−3.2	Manzamine A	Alkaloid	32.05 549.359
21	22.5	312.29	311.31	C_20_H_40_O_2_	0.7	Methylnonadecan-1-oate	Fatty acid	57.072, 81.071, 95.084, 97.101, 123.119, 137.133, 151.146, 278.231, 313.252

r.t, retention time; error ppm, ±5.

Furthermore, the HAECA was evaluated for polyphenolic quantification by HPLC-PDA. A total of 23 polyphenolic standards were applied in these experiments. The presence of the compounds was confirmed by comparing the retention time and UV/Vis spectra to the standard compounds. The polyphenols present in HAECA are listed in Table 3. Gentisic acid was the dominant (2.91 ± 1.23 μg/mg DE) compound, followed by cinnamic acid (2.07 ± 1.2 μg/mg DE). Chlorogenic acid levels were found below the detection limit. Significant amounts of other polyphenols, conidendrine, and skullcapflavone, were also identified. These compounds might explain the anti-inflammatory potential of HAECA based on previous reports of the significant anti-inflammatory effect of polyphenols (Basit et al., 2022b).

**TABLE 3:** Polyphenolic compounds quantified in HAECA.

S. No.	Standard name	Polyphenols quantified (µg/mg DE)
1	Syringic acid	0.17 ± 0.13
2	Kaempferol	N.D.
3	Caffeic acid	N.D.
4	Cinnamic acid	2.07 ± 1.2
5	Vannilic acid	N.D.
6	Gentisic acid	2.91 ± 1.23
7	Ferulic acid	N.D.
8	Skullcapflavone	0.96 ± 1.03
9	Rutin	N.D.
10	Apigenin	N.D.
11	Conidendrine	1.73 ± 1.02
12	Naringenin	N.D.
13	Catechin	N.D.
14	Chlorogenic acid	BLD

All the values are expressed as mean ± standard deviation (std) (*n* = 3). N.D., not detected; DE, dry extract; BLD, below limit of detection (<0.1 μg/g DE).

### 3.2 Toxicity profiling

#### 3.2.1 *In vitro* cytotoxicity using MTT assays

**FIGURE 4:**
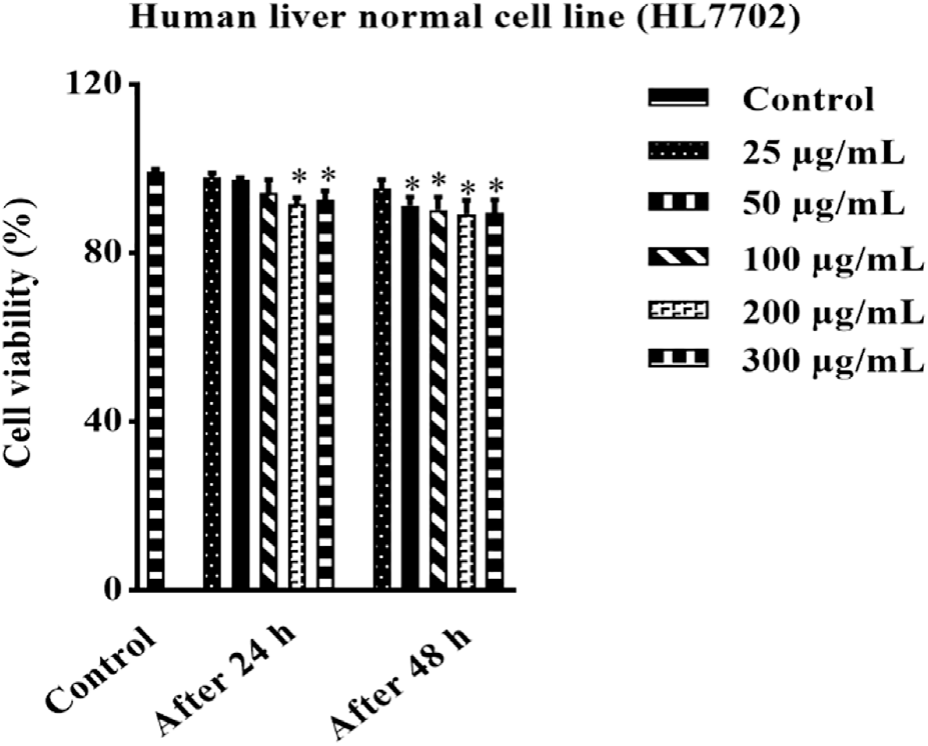
Effect of HAECA on human normal liver cells.

The effect of HAECA at the cellular level was studied through *in vitro* MTT assays. Human normal liver cells were used to assess the toxicity of HAECA. The use of the MTT assay for the safety profiling of plant extracts has increased in recent years. The findings revealed the non-toxic nature of the extract by showing the least effect on the percent cell viability of liver normal cells (Figure 4). The controls in this assay were untreated cells. The cell viability study of the extract can be extended to other cell lines to get more in-depth insights into the toxicity of the extract.

### 3.3 *In vitro* biological profiling

#### 3.3.1 Anticancer efficacy of HAECA

**FIGURE 5:**
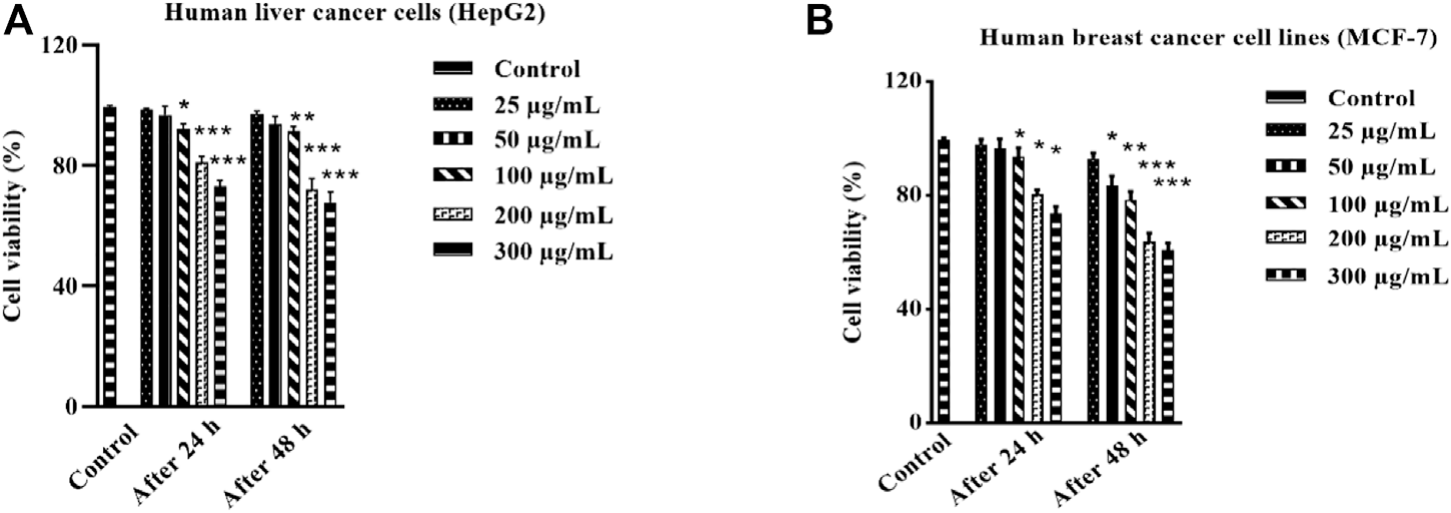
Anticancer effect of HAECA on human liver cancer cells **(A)** and cell viability (%) against human breast cancer cells **(B)**. All values are expressed as mean ± SD (*n* = 3).

The investigation of anticancer properties of unexplored plant species is of prime importance in the field of biomedical applications. The anticancer evaluation of HAECA was first determined in *in vitro* MTT assays against human breast cancer (MCF-7) and human liver cancer (HepG2) lines. The extract was used at different concentrations (300, 200, 100, 50, and 25 μg/ml) to evaluate the dose-dependent effect of HAECA. The anticancer effects on these cell lines were assessed after 24 and 48 h. The results are displayed in Figures 5A, B which demonstrated the low to moderate anticancer activity of HAECA. As shown in (Figures 5A, B) the extract showed statistically significant decreases in percent cell viability compared to the control group after 24 h with maximum effects (*p* < 0.001) observed at 200 and 300 μg/ml. Similarly, HAECA showed a significant anticancer effect: *p* < 0.01 at 50 μg/ml and *p* < 0.001 at 200 and 300 μg/ml each on both cell lines. The IC_50_ values of HAECA in HepG2 cells were 559.12 and 451.15 μg/ml after 24 h and 48 h respectively. Similarly, the IC_50_ values of HAECA in MCF-7 cells were 605.94 and 368.65 μg/ml after 24 and 48 h respectively. The anticancer effect of the extract might be due to the modulation of inflammatory markers, which have previously been shown to ameliorate the effect on cancer cells (Assaf et al., 2013).

#### 3.3.2 Antioxidant potential of HAECA

**FIGURE 6:**
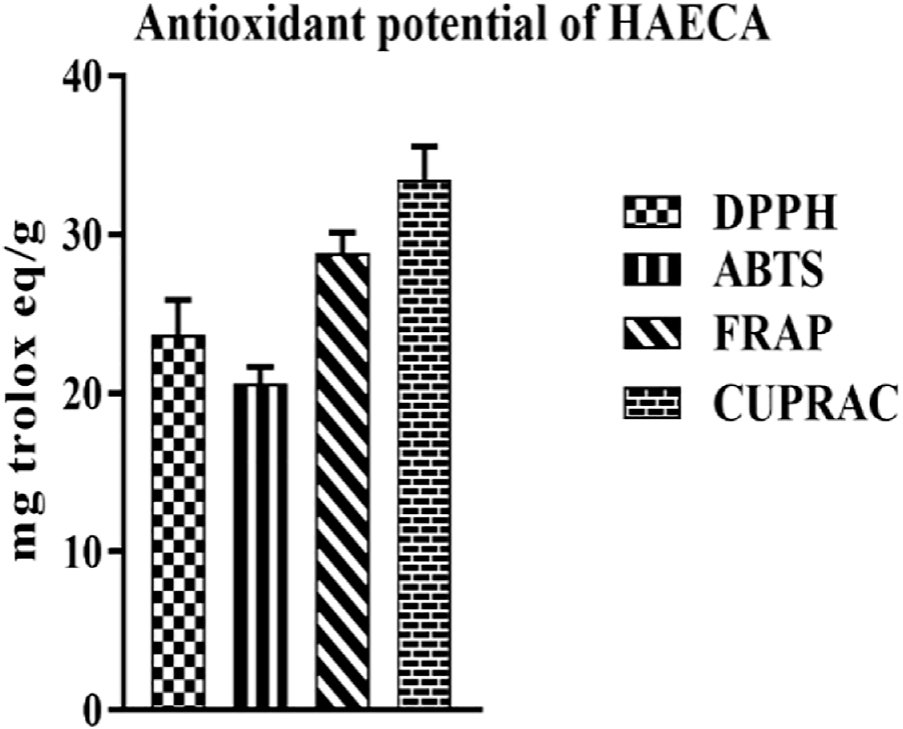
Antioxidant potential of HAECA.

The free radicals mediated the etiological processes of various diseases. The generation of free radicals initiates and propagates toxic reactions such as lipid peroxidation, which, in turn, lead to the fragmentation of macromolecules and cell death (Umamaheswari and Chatterjee, 2008). The effects of these reactive oxygen species (ROS) are diminished by endogenous or exogenous antioxidants (Fang et al., 2002). Plant secondary metabolites such as phenols and flavonoids act as strong antioxidants. Moreover, some endogenous antioxidant enzymes such as catalase, SOD, and GSH may ameliorate health pathologies (Lee et al., 2004). The current study applied four different methods to evaluate the antioxidant properties of HAECA. The highest activity was observed in the CUPRAC method (33.45 ± 1.3 mg TE/g DE) followed by the FRAP assay (28.81 ± 0.34 mg TE/g DE). Similarly, in the DPPH assay HAECA showed 23.66 ± 2.3 mg TE/g DE activity (Figure 6). The good antioxidant activity may be due to the presence of flavonoids and phenols in HAECA, as previous studies correlated high antioxidant activity to high levels of phenols and flavonoids (Ahmad et al., 2019; Mondal et al., 2019).

### 3.4 Anti-inflammatory effects of HAECA

#### 3.4.1 Effects of HAECA on carrageenan paw-induced inflammation

**FIGURE 7:**
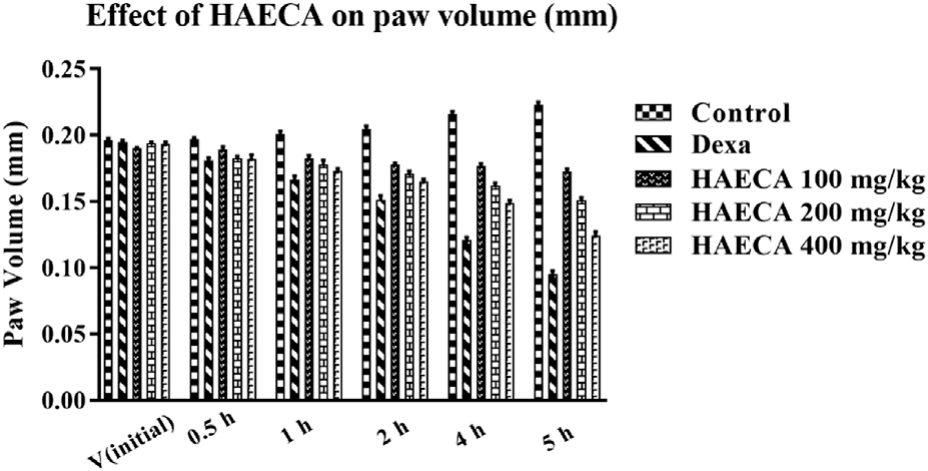
Effect of HAECA on carrageenan-induced paw edema in mice.*p < 0.05, **p < 0.01, ***p < 0.001 compared to the control group. *ANOVA* (one-way) followed by *post hoc* analysis (Tukey’s multiple comparison test).

**FIGURE 8:**
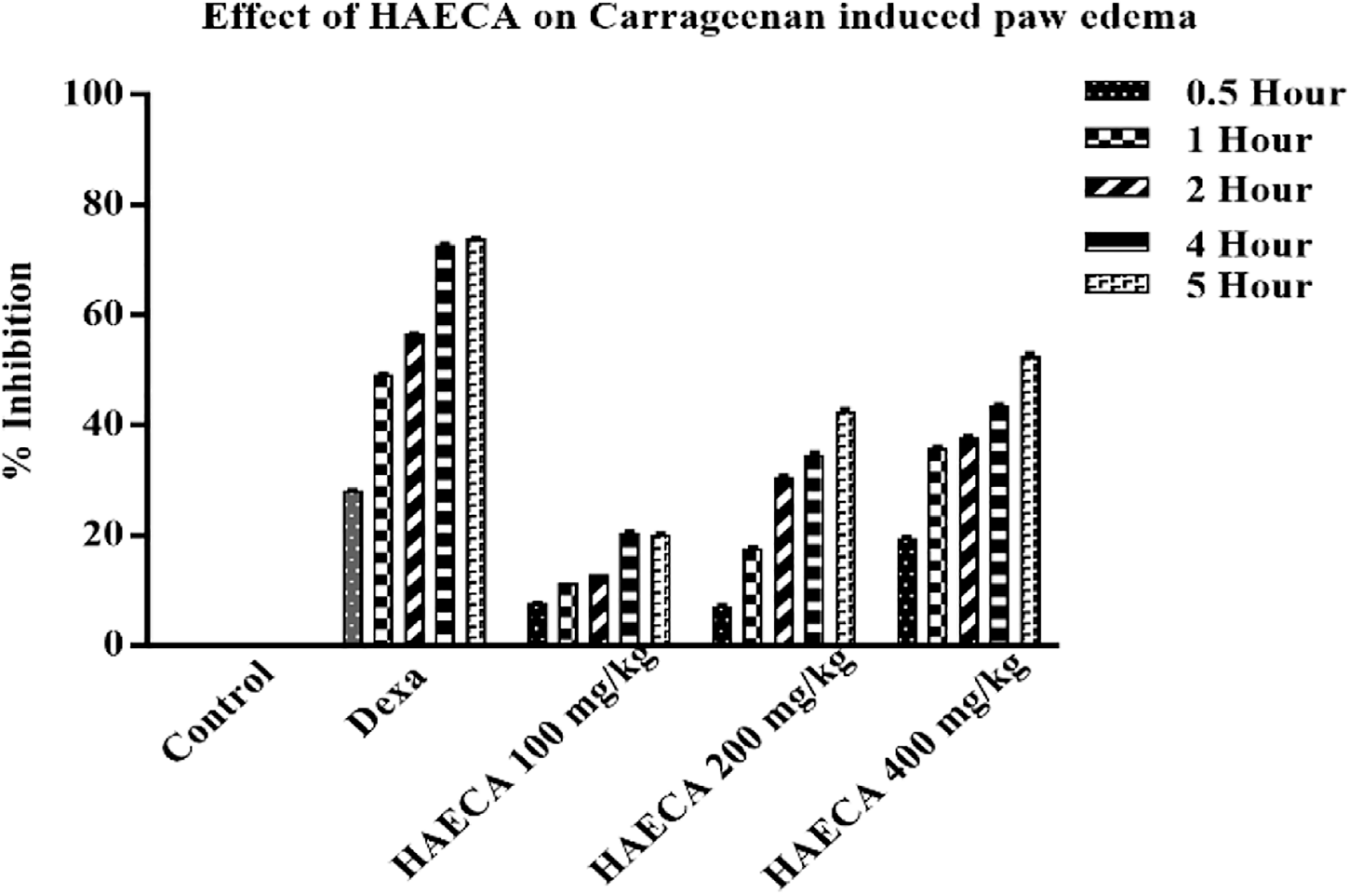
Percent inhibition of carrageenan-induced paw edema by HAECA.

The evaluation of the acute anti-inflammatory effect of natural products is one of the most common approaches in practice (Piva et al., 2021). Carrageenan induces inflammation primarily in two phases. The main feature of phase I is the release of kinin, cyclooxygenase, leukotriene, and histamine. Phase I is the predominant phase and lasts from 1 to 1.5 hours. The first phase is followed by the second phase, which features the production of prostaglandins (Ayal et al., 2019). Carrageenan administration in the present study caused a marked increase in the mouse paw volume (Table 4), consistent with previous reports (Piva et al., 2021). The induced inflammation was lower in extract-treated animals. As shown in Table 4 and Figure 7, the extract showed maximum results and the highest percent inhibition (*p* < 0.001) at 400 mg/kg (*p* < 0.01) at 200 mg/kg at 5 h after carrageenan injection (Figure 8). Animals treated with standard dexamethasone (75 mg/kg) showed significant inhibition (*p* < 0.05, *p* < 0.01, *p* < 0.001, *p* < 0.001, and *p* < 0.001, respectively) at 0.5, 1, 2, 4, and 5 h after injection compared to the control group, which was consistent with previous reports (Piva et al., 2021). Overall, the extract showed dose and time-dependent anti-inflammatory effects. The results showed that HAECA significantly inhibited edema in the later stage, which occurs after 2–6 h. This inhibition of edema in the second phase by HAECA might be due to the suppression of prostaglandins by inhibiting cyclooxygenase and its related products, as also confirmed by the results of the *in silico* molecular docking study.

**TABLE 4:** Effect of HAECA on carrageenan-induced paw edema volume (cm).

Paw volume (cm), mean ± SD.						
Group	Initial volume	0.5 h	1 h	2 h	4 h	5 h
Normal saline (1 ml/kg)	0.196 ± 0.32	0.197 ± 0.32	0.201 ± 0.16	0.205 ± 0.83	0.216 ± 0.57	0.221 ± 0.32
Dexamethasone 75 mg/kg	0.193 ± 0.41	0.181 ± 0.12 *	0.167 ± 0.04 **	0.151 ± 0.17*	0.123 ± 0.26 *	0.098 ± 0.23
HAECA 100 mg/kg	0.189 ± 0.21	0.190 ± 0.24	0.182 ± 0.32	0.179 ± 0.56	0.179 ± 1.73	0.172 ± 0.41
HAECA 200 mg/kg	0.193 ± 0.09	0.184 ± 0.05	0.178 ± 0.45	0.171 ± 0.46 *	0.162 ± 0.51	0.153 ± 0.29 **
HAECA 400 mg/kg	0.194 ± 1.21	0.183 ± 0.27	0.173 ± 0.42	0.165 ± 0.71 *	0.149 ± 0.61 **	0.127 ± 0.75 ***

All values are presented as mean ± SD (*n* = 3). *p < 0.05, **p < 0.01, and ***p < 0.001 compared to the control group.

#### 3.4.2 Effects of HAECA on oxidative stress and inflammatory markers

**FIGURE 9:**
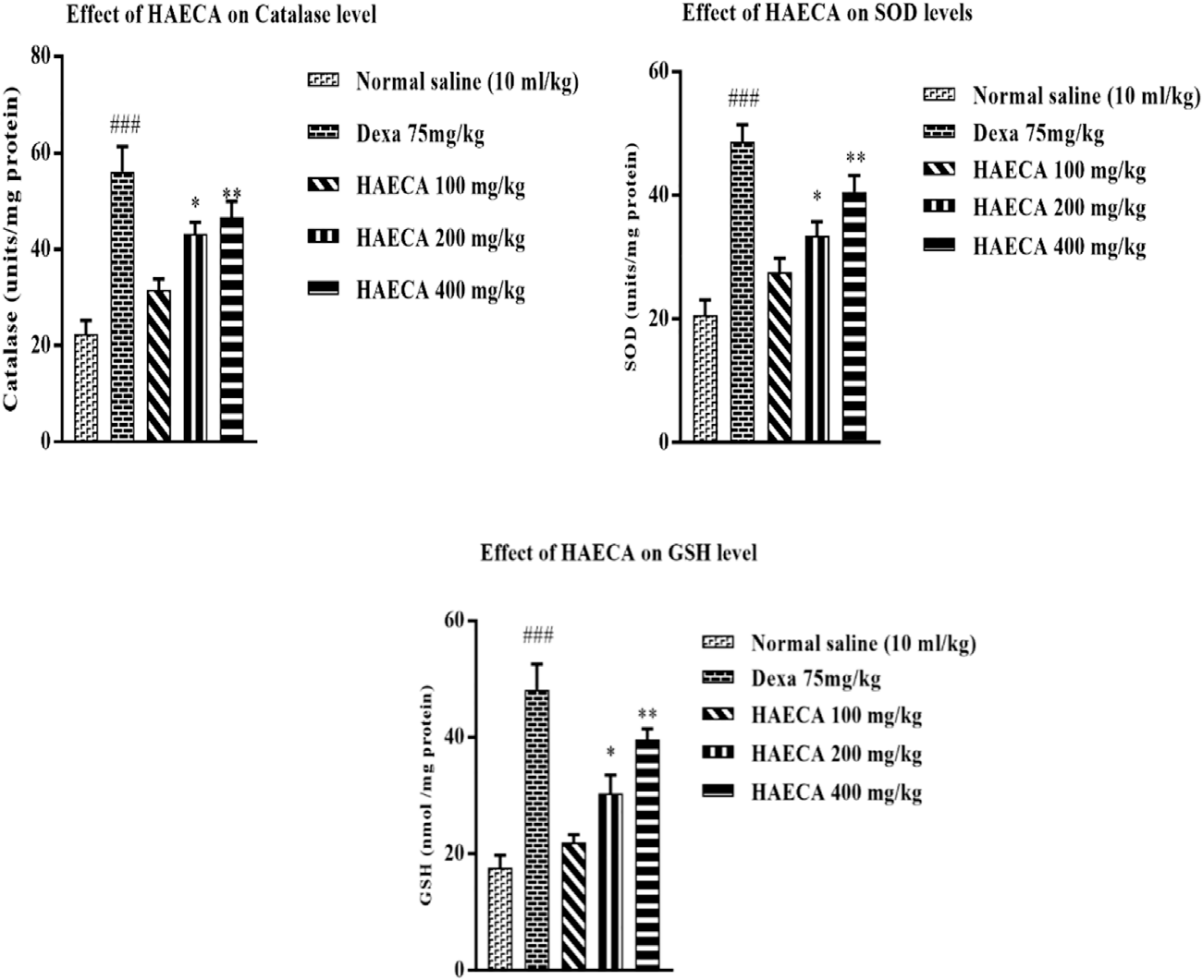
Effect of HAECA on oxidative stress markers. ###*p* < 0.001 compared to the negative control; *p < 0.05 compared to the control group; and **p < 0.01, ***p < 0.001 compared to the control. All values are expressed as mean ± SD., (*n* = 3).

Prostaglandin production leads to the initiation of ROS generation and the downregulation of oxidative stress markers, the underlying causes of inflammation (Ansari et al., 2020; Ouda et al., 2021). As HAECA displayed excellent anti-inflammatory activity, it was also assayed to estimate the levels of oxidative stress markers in mice paw tissue to elucidate the possible mechanism involved in its anti-inflammatory effect. The oxidative stress markers included catalase (CAT), SOD, and GSH. The findings revealed a maximum effect (*p* < 0.01) in terms of increased levels of catalase, SOD, and GSH in HAECA-treated groups at 400 mg/kg compared to the control group (Figure 9). These results suggested that the anti-inflammatory effect of HAECA may be due to increased levels of oxidant stress markers.

**FIGURE 10:**
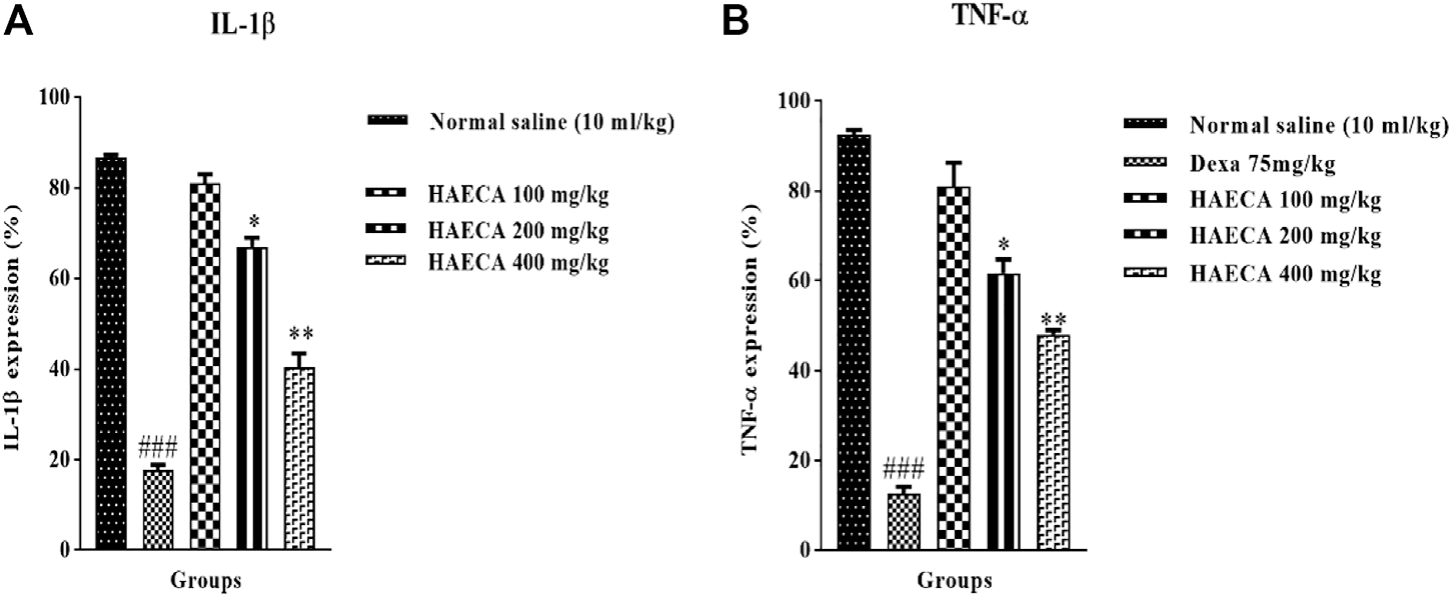
Effect of HAECA on IL-1β **(A)** and TNF-α **(B)** expression. *p < 0.05, **p < 0.01, ***p < 0.001 compared to the standard. ### *p* < 0.001 compared to the normal saline group.

The involvement of IL-1β and TNF-α in inflammation initiation and maintenance is well established. These markers are considered the front-line contributors to inflammation, with both direct and indirect effects on the etiology of inflammation. In the indirect effect, they trigger the production of prostaglandins and other inflammatory mediators (Ansari et al., 2020). Carrageenan-induced inflammation involves the release of IL-1β and TNF-α, as also observed in the current study (Mansouri et al., 2015). The levels of IL-1β and TNF-α were quantified to elucidate the possible anti-inflammatory mechanisms of HAECA. HAECA (400 mg/kg) led to a significant (*p* < 0.01) decrease in TNF-α levels compared to the control group. Similarly, 400 mg/kg of HAECA showed the highest (*p* < 0.01) decrease in IL-1β levels compared to those in the control group. The positive control dexamethasone also showed a maximum decrease (*p* < 0.001) in IL-1β and TNF-α levels at 1 mg/kg (Figure 10). The polyphenolic compounds in HAECA might be the triggering force behind its anti-inflammatory potential. The roles of polyphenolic compounds and alkaloids are evident from previous reports (Ammar et al., 2018). The phytochemical composition of HAECA revealed the tentative identification of many anti-inflammatory flavonoids such as skullcapflavone and conidendrine, phenols including syringic acid and sinapaldehyde, and alkaloids and lignans which were demonstrated to be significant anti-inflammatory agents in previous investigations (Moon et al., 2006; Aggarwal et al., 2013; Conti et al., 2013).

### 3.5 *In silico* studies

**FIGURE 11:**
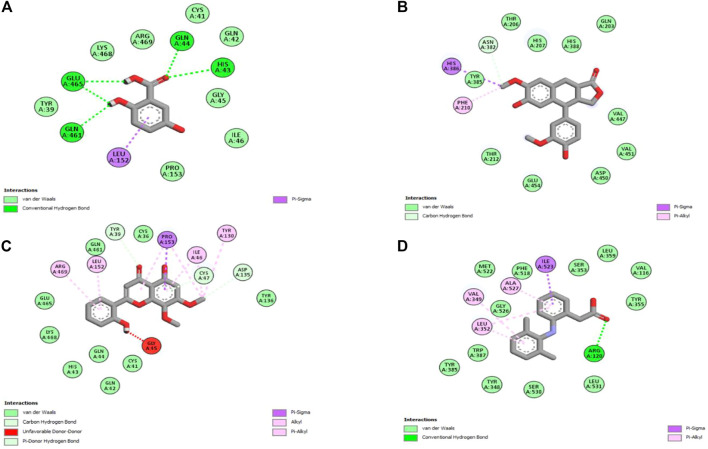
Interaction of the major compounds with cyclooxygenase-I. **(A)** Gentisic acid; **(B)** conidendrine; **(C)** skullcapflavone; and **(D)** diclofenac.

**FIGURE 12:**
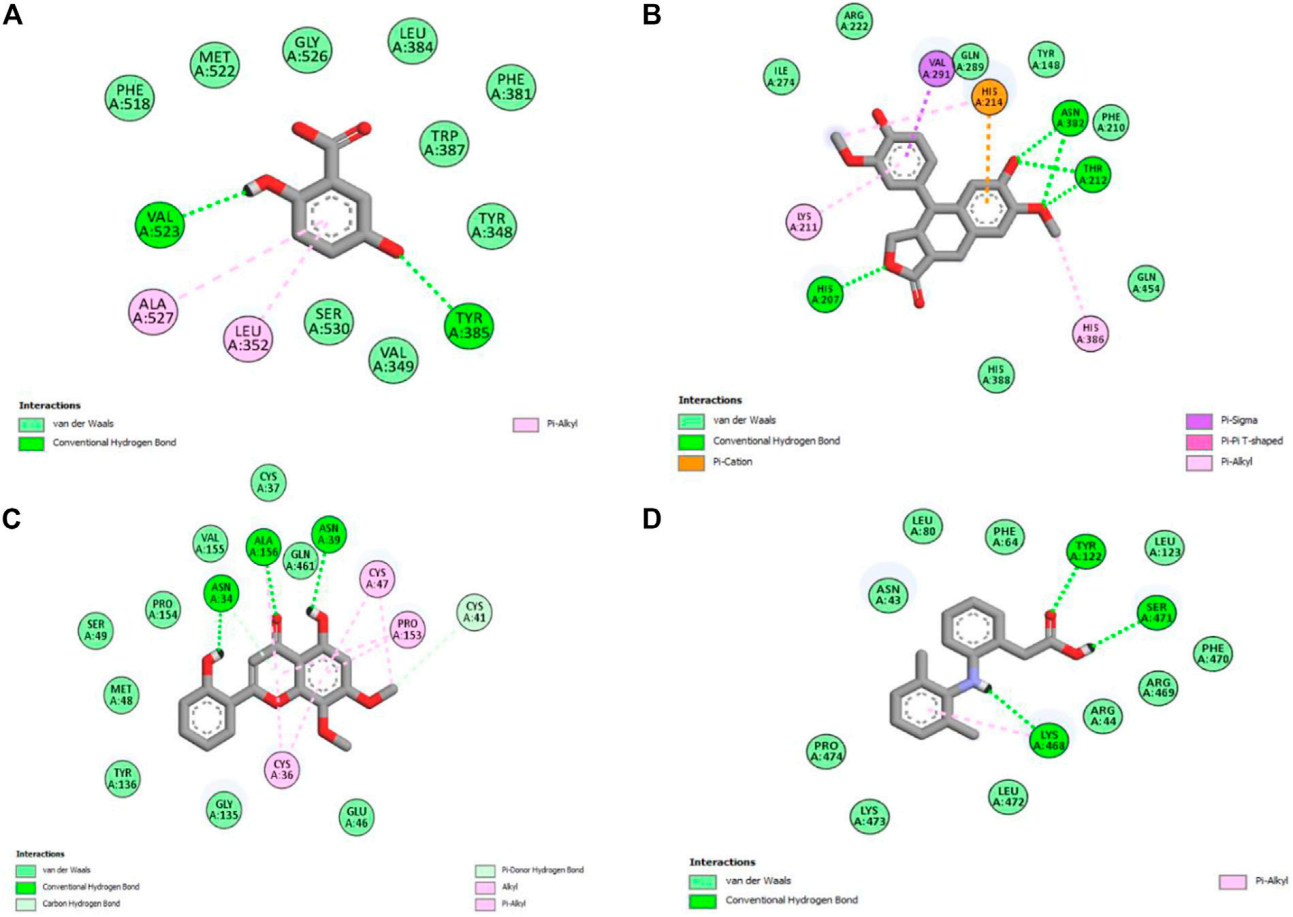
Interaction of the major compounds with cyclooxygenase-II. **(A)** Gentisic acid; **(B)** conidendrine; **(C)** skullcapflavone; and **(D)** diclofenac.

Molecular docking is one of the most widely employed approaches for the prediction of binding energies of phytoconstituents and the correlation of pharmacological activities. The roles of COX-I and COX-II in inflammation initiation are established. HAECA showed a promising anti-inflammatory effect in the carrageenan-induced paw edema model. Therefore, to provide insights into this effect, three compounds identified in the UPLC-Q-TOF-MS profiling of HAECA were subjected to *in silico* docking studies against COX-I and COX-II (Table 5). Among the studied compounds, skullcapflavone showed high binding affinities with both COX-I and COX-II compared to the standard compound diclofenac. The detailed interactions of the amino acid residues and the compounds are displayed in Figures 11, 12. The good docking score showed by the compounds against the tested compounds indicated that the anti-inflammatory effect of HAECA might be due to their inhibitory effects on COX-I and COX-II.

**TABLE 5:** Details of binding affinities and interacting amino acid residues.

Cyclooxygenase-I		
Compound name	Binding affinity	Interacting amino acids
Gentisic acid	−6.2	Tyr39, Cys41, Gln42, His43, Gln44, Gly45, Ile46, Leu152, Pro153, Gln461, Glu465, Lys468, and Arg469
Skullcapflavone	−8.6	Cys36, Tyr39, Cys41, Gln42, His43, Gln44, Ile46, Cys47, Tyr130, Asp135, Pro153, Leu152, Gln461, Glu465, Lys468, and Arg469
Conidendrine	−7.1	Gln203, Thr206, His207, Phe219, Thr212, Asn382, Tyr385, His386, His388, Val447, Asp450, Val451, and Glu454
Diclofenac	−7.3	Val116, Arg120, Tyr348, Val349, Leu352, Ser353, Tyr355, Leu359, Tyr385, Trp387, Phe518, Met522, Ile523, Gly526, Ser530, and Leu531
Cyclooxygenase-II		
Gentisic acid	−6.2	Tyr348, Val349, Leu352, Phe381, Leu384, Tyr385, Trp387, Phe518, Met522, Gly526, Ala527, and Ser530
Skullcapflavone	−8.5	Asn34, Cys36, Cys37, Asn39, Cys41, Glu46, Cys47, Met48, Ser49, Gly135, Tyr136, Pro153, Pro154, Val155, Ala156, and Gln461
Conidendrine	−8.3	Tyr148, His207, Phe210, Lys211, Thr212, Arg222, Ile274, Gln289, Val291, Asn382, His386, His388, and Gln454
Diclofenac	−6.6	Asn43, Arg44, Phe64, Tyr122, Leu123, Lys468, Arg469, Phe470, Ser471, and Leu472

*HB, hydrogen bonding.

## 4 Conclusion

This study investigated the anti-inflammatory, antioxidant, cytotoxic, and phytochemical profiling of the hydroalcoholic extract of *C. amara.* The findings demonstrated a significant inhibitory effect of HAECA on late-phase carrageenan-induced paw edema, which may be due to the modulatory effect of the extract on oxidative stress and inflammatory markers. Moreover, the good interaction score of the selected compounds against COX-I and COX-II also supports the possible anti-inflammatory mechanism of HAECA. The extract showed good *in vitro* antioxidant potential, which may be correlated with the presence of phenols, flavonoids, alkaloids, and lignans revealed in UPLC-Q-TOF-MS profiling of HAECA. The *in vitro* cytotoxicity studies showed no effect on normal cell lines and moderate to low effects on the cancer cell lines. Overall, these results suggest that the species is non-toxic and paves the way for investigations to isolate and study the phytoconstituents at subacute levels.

## Data Availability

The original contributions presented in the study are included in the article/Supplementary Material. Further inquiries can be directed to the corresponding authors.

## References

[B1] Aggarwal M. Kondeti B. Mckenna R. (2013). Anticonvulsant/antiepileptic carbonic anhydrase inhibitors: A patent review. Expert Opin. Ther. Pat. 23, 717–724. 23514045 10.1517/13543776.2013.782394

[B2] Ahmad I. Ahmed S. Akkol E. K. Rao H. Shahzad M. N. Shaukat U. (2022). GC–MS profiling, phytochemical and biological investigation of aerial parts of Leucophyllum frutescens (Berl.) IM Johnst (Cenizo). South Afr. J. Bot. 148, 200–209. 10.1016/j.sajb.2022.04.038

[B3] Ahmad S. Hassan A. Rehman T. Basit A. Tahir A. Arshad M. A. (2019). *In vitro* bioactivity of extracts from seeds of Cassia absus L. growing in Pakistan. J. Herb. Med. 16, 100258–100265. 10.1016/j.hermed.2019.100258

[B4] Ammar I. Salem M. B. Harrabi B. Mzid M. Bardaa S. Sahnoun Z. (2018). Anti-inflammatory activity and phenolic composition of prickly pear (Opuntia ficus-indica) flowers. Ind. Crops. Prod. 112, 313–319. 10.1016/j.indcrop.2017.12.028

[B5] Ansari M. Y. Ahmad N. Haqqi T. M. (2020). Oxidative stress and inflammation in osteoarthritis pathogenesis: Role of polyphenols. Biomed. Pharmacother. 129, 110452. 10.1016/j.biopha.2020.110452 32768946 PMC8404686

[B6] Asmat U. Abad K. Ismail K. (2016). Diabetes mellitus and oxidative stress—a concise review. Saudi Pharm. J. 24, 547–553. 10.1016/j.jsps.2015.03.013 27752226 PMC5059829

[B7] Assaf A. M. Haddadin R. N. Aldouri N. A. Alabbassi R. Mashallah S. Mohammad M. (2013). Anti-cancer, anti-inflammatory and anti-microbial activities of plant extracts used against hematological tumors in traditional medicine of Jordan. J. Ethnopharmacol. 145, 728–736. 10.1016/j.jep.2012.11.039 23246454

[B8] Ayal G. Belay A. Kahaliw W. (2019). Evaluation of wound healing and anti-inflammatory activity of the leaves of Calpurnia aurea (Ait.) Benth (fabaceae) in mice. Wound Med. 25, 100151. 10.1016/j.wndm.2019.100151

[B9] Babu A. Prasanth K. Balaji B. (2015). Effect of curcumin in mice model of vincristine-induced neuropathy. Pharm. Biol. 53, 838–848. 10.3109/13880209.2014.943247 25429779

[B10] Balkrishna A. Verma S. Sharma P. Tomer M. Srivastava J. Varshney A. (2021). Comprehensive and rapid quality evaluation method for the ayurvedic medicine divya-swasari-vati using two analytical techniques: UPLC/QToF MS and HPLC–DAD. Pharmaceuticals 14, 297. 10.3390/ph14040297 33801579 PMC8067215

[B11] Basit A. Ahmad S. Naeem A. Usman M. Ahmed I. Shahzad M. N. (2021). Chemical profiling of Justicia vahlii Roth.(Acanthaceae) using UPLC-QTOF-MS and GC-MS analysis and evaluation of acute oral toxicity, antineuropathic and antioxidant activities. J. Ethnopharmacol. 287, 114942. 10.1016/j.jep.2021.114942 34968664

[B12] Basit A. Ahmad S. Naeem A. Usman M. Ahmed I. Shahzad M. N. (2022a). Chemical profiling of Justicia vahlii Roth.(Acanthaceae) using UPLC-QTOF-MS and GC-MS analysis and evaluation of acute oral toxicity, antineuropathic and antioxidant activities. J. Ethnopharmacol. 287, 114942. 10.1016/j.jep.2021.114942 34968664

[B13] Basit A. Ahmad S. Sherif A. E. Ati H. Y. Ovatlarnporn C. Khan M. A. (2022b). New mechanistic insights on Justicia vahlii roth: UPLC-Q-TOF-MS and GC-MS based metabolomics, *in-vivo*, *in-silico* toxicological, antioxidant based anti-inflammatory and enzyme inhibition evaluation. Arabian J. Chem. 15 (10), 104135.

[B14] Brito T. Lima L. Santos M. Moreira R. Cameron L. Fai A. (2021). Antimicrobial, antioxidant, volatile and phenolic profiles of cabbage-stalk and pineapple-crown flour revealed by GC-MS and UPLC-MSE. Food Chem. 339, 127882–127911. 10.1016/j.foodchem.2020.127882 32889131

[B15] Chanda J. Mukherjee P. K. Biswas R. Biswas S. Tiwari A. K. Pargaonkar A. (2019). UPLC‐QTOF‐MS analysis of a carbonic anhydrase‐inhibiting extract and fractions of Luffa acutangula (L.) Roxb (ridge gourd). Phytochem. Anal. 30, 148–155. 10.1002/pca.2800 30402952

[B16] Chavan R. R. Bhinge S. D. Bhutkar M. A. Randive D. S. Wadkar G. H. Todkar S. S. (2020). Characterization, antioxidant, antimicrobial and cytotoxic activities of green synthesized silver and iron nanoparticles using alcoholic Blumea eriantha DC plant extract. Mater. Today Commun. 24, 101320. 10.1016/j.mtcomm.2020.101320

[B17] Conti P. Varvara G. Murmura G. Tete S. Sabatino G. Saggini A. (2013). Comparison of beneficial actions of non-steroidal anti-inflammatory drugs to flavonoids. J. Biol. Regul. Homeost. Agents 27, 1–7. 23489682

[B18] Cunha A. G. Brito E. S. Moura C. F. Ribeiro P. R. Miranda M. R. A. (2017). UPLC–qTOF-MS/MS-based phenolic profile and their biosynthetic enzyme activity used to discriminate between cashew apple (Anacardium occidentale L.) maturation stages. J. Chromatogr. B 1051, 24–32. 10.1016/j.jchromb.2017.02.022 28285020

[B19] Dilshad R. Ahmad S. Aati H. Y. Al-Qahtani J. H. Sherif A. E. Hussain M. (2022). Phytochemical profiling, *in vitro* biological activities, and *in-silico* molecular docking studies of Typha domingensis. Arabian J. Chem. 15, 104133. 10.1016/j.arabjc.2022.104133

[B20] Doherty V. Ogunkuade O. Kanife U. (2010). Biomarkers of oxidative stress and heavy metal levels as indicators of environmental pollution in some selected fishes in Lagos, Nigeria. Am. Eurasian J. Agric. Environ. Sci. 7, 359–365.

[B21] Duan S. G. Hong K. Tang M. Tang J. Liu L. X. Gao G. F. (2021). Untargeted metabolite profiling of petal blight in field-grown Rhododendron agastum using GC-TOF-MS and UHPLC-QTOF-MS/MS. Phytochemistry 184, 112655. 10.1016/j.phytochem.2021.112655 33540237

[B22] Elshamy A. I. Farrag A. R. H. Ayoub I. M. Mahdy K. A. Taher R. F. Gendy A. E.-N. G. (2020). UPLC-qTOF-MS phytochemical profile and antiulcer potential of Cyperus conglomeratus Rottb. alcoholic extract. Molecules 25, 4234. 10.3390/molecules25184234 32942704 PMC7570889

[B23] Fang Y.-Z. Yang S. Wu G. (2002). Free radicals, antioxidants, and nutrition. Nutrition 18, 872–879. 10.1016/s0899-9007(02)00916-4 12361782

[B24] Farombi E. Adelowo O. Ajimoko Y. (2007). Biomarkers of oxidative stress and heavy metal levels as indicators of environmental pollution in African cat fish (*Clarias gariepinus*) from Nigeria Ogun River. Int. J. Environ. Res. Public health 4, 158–165. 10.3390/ijerph2007040011 17617680 PMC3728582

[B25] Fattahi A. Shakeri A. Tayarani Najaran Z. Kharbach M. Segers K. Heyden Y. V. (2021). UPLC–PDA‐ESI–QTOF–MS/MS and GC-MS analysis of Iranian Dracocephalum moldavica L. Food Sci. 9, 4278–4286. 10.1002/fsn3.2396 PMC8358350 34401078

[B26] Hanhineva K. Rogachev I. Aura A. M. Aharoni A. Poutanen K. Mykkänen H. (2012). Identification of novel lignans in the whole grain rye bran by non-targeted LC–MS metabolite profiling. Metabolomics 8, 399–409. 10.1007/s11306-011-0325-0

[B27] Hu L. Xiong Y. Zou Z. Yang Y. He J. Zhong L. (2020). Identifying the chemical markers in raw and wine-processed Scutellaria baicalensis by ultra‐performance liquid chromatography/quadrupole time‐of‐flight mass spectrometry coupled with multiple statistical strategies. Biomed. Chromatogr. 34, 48499–e4914. 10.1002/bmc.4849 32302414

[B28] Jones D. P. (2006). Redefining oxidative stress. Antioxidants redox Signal. 8, 1865–1879. 10.1089/ars.2006.8.1865 16987039

[B29] Kakkar P. Das B. Viswanathan P. (1984). A modified spectrophotometric assay of superoxide dismutase. Int. J. Biol. Biotechnol. 21, 130–132. 6490072

[B30] Khan A. M. Khan A. U. Ali H. Islam S. U. Seo E. K. Khan S. (2020). Continentalic acid exhibited nephroprotective activity against the LPS and E. coli-induced kidney injury through inhibition of the oxidative stress and inflammation. Int. Immunopharmacol. 80, 106209. 10.1016/j.intimp.2020.106209 32004924

[B31] Khan A. Ullah M. Z. Afridi R. Rasheed H. Khalid S. Ullah H. (2019). Antinociceptive properties of 25‐methoxy hispidol A, a triterpinoid isolated from Poncirus trifoliata (Rutaceae) through inhibition of NF-κB signalling in mice. Phytotherapy Res. 33, 327–341. 10.1002/ptr.6223 30456885

[B32] Lee J. Koo N. Min D. B. (2004). Reactive oxygen species, aging, and antioxidative nutraceuticals. Compr. Rev. Food Sci. Food Saf. 3, 21–33. 10.1111/j.1541-4337.2004.tb00058.x 33430557

[B33] Li C. Huang C. Lu T. Wu L. Deng S. Yang R. (2014). Tandem mass spectrometric fragmentation behavior of lignans, flavonoids and triterpenoids in Streblus asper. Rapid Commun. Mass Spectrom. 28, 2363–2370. 10.1002/rcm.7035 25279750

[B34] Lihová J. Marhold K. Kudoh H. Koch M. A. (2006). Worldwide phylogeny and biogeography of Cardamine flexuosa (Brassicaceae) and its relatives. Am. J. Bot. 93, 1206–1221. 10.3732/ajb.93.8.1206 21642185

[B35] Lihová J. Marhold K. Neuffer B. J. T. (2000). Taxonomy of Cardamine amara (cruciferae) in the iberian peninsula. Taxon 49, 747–763. 10.2307/1223975

[B36] Luczaj L. (2012). Ethnobotanical review of wild edible plants of Slovakia. Acta Soc. Bot. Pol. 81, 245–255. 10.5586/asbp.2012.030

[B37] Ma’arif B. Suryadinata A. Laswati H. Agil M. (2018). Metabolite profiling of 96% ethanol extract from Marsilea crenata Presl. leaves using UPLC-QToF-MS/MS and anti-neuroinflammatory predicition activity with molecular docking. J. Trop. Pharm. Chem. 4 (6), 261–270.

[B38] Mansouri M. T. Hemmati A. A. Naghizadeh B. Mard S. A. Rezaie A. Ghorbanzadeh B. (2015). A study of the mechanisms underlying the anti-inflammatory effect of ellagic acid in carrageenan-induced paw edema in rats. Indian J. Pharmacol. 47, 292. 10.4103/0253-7613.157127 26069367 PMC4450555

[B39] Mondal M. Hossain M. M. Rahman M. A. Saha S. Uddin N. Hasan M. R. (2019). Hepatoprotective and antioxidant activities of Justicia gendarussa leaf extract in carbofuran-induced hepatic damage in rats. Chem. Res. Toxicol. 32, 2499–2508. 10.1021/acs.chemrestox.9b00345 31696704

[B40] Montaut S. Bleeker R. S. (2013). Review on Cardamine diphylla (Michx.) A. wood (Brassicaceae): Ethnobotany and glucosinolate chemistry. J. Ethnopharmacol. 149, 401–408. 10.1016/j.jep.2013.07.020 23892204

[B41] Moon Y. J. Wang X. Morris M. E. (2006). Dietary flavonoids: Effects on xenobiotic and carcinogen metabolism. Toxicolo. vitro 20, 187–210. 10.1016/j.tiv.2005.06.048 16289744

[B42] Ouda A. N. Fatiha M. Sadia M. Zohra S. F. Noureddine D. (2021). *In vivo* anti-inflammatory activity of aqueous extract of carthamus caeruleus L rhizome against carrageenan-induced inflammation in mice. Jordan J. Biol. Sci. 14, 529–535.

[B43] Pieczykolan A. Pietrzak W. Gawlik-Dziki U. Nowak R. (2021). Antioxidant, anti-inflammatory, and anti-diabetic activity of phenolic acids fractions obtained from aerva lanata (L.) juss. Molecules 26, 3486. 10.3390/molecules26123486 34201147 PMC8228310

[B44] Piva R. C. Verdan M. H. Branquinho L. S. Kassuya C. a. L. Cardoso C. a. L. (2021). Anti-inflammatory activity and chemical composition of aqueous extract and essential oil from leaves of Ocimum selloi Benth. J. Ethnopharmacol. 275, 114136. 10.1016/j.jep.2021.114136 33892069

[B45] Qi Y. Li S. Pi Z. Song F. Lin N. Liu S. (2014). Chemical profiling of Wu-tou decoction by UPLC–Q-TOF-MS. Talanta 118, 21–29. 10.1016/j.talanta.2013.09.054 24274266

[B46] Raza A. Hafeez M. B. Zahra N. Shaukat K. Umbreen S. Tabassum J. (2020). “The plant family Brassicaceae: Introduction, biology, and importance,” in The plant family brassicaceae (Singapore: Springer), 1–43.

[B47] Shahzad M. N. Ahmad S. Tousif M. I. Ahmad I. Rao H. Ahmad B. (2022). Profiling of phytochemicals from aerial parts of Terminalia neotaliala using LC-ESI-MS2 and determination of antioxidant and enzyme inhibition activities. PloS one 17, e0266094. 10.1371/journal.pone.0266094 35358239 PMC8970405

[B48] Tandon R. Khanna R. Dorababu M. Goel R. (2004). Oxidative stress and antioxidants status in peptic ulcer and gastric carcinoma. J. Physiol. Pharmacol. 48, 115–118. 15270379

[B49] Umamaheswari M. Chatterjee T. (2008). *In vitro* antioxidant activities of the fractions of Coccinia grandis L. leaf extract. Afr. J. Tradit. Complement. Altern. Med. 5, 61–73. PMC2816591 20162057

[B50] Uysal S. Ugurlu A. Zengin G. Baloglu M. C. Altunoglu Y. C. Mollica A. (2018). Novel *in vitro* and *in silico* insights of the multi-biological activities and chemical composition of Bidens tripartita L. Food Chem. Toxicol. 111, 525–536. 10.1016/j.fct.2017.11.058 29217268

[B51] Xu T. Yang M. Li Y. Chen X. Wang Q. Deng W. (2013). An integrated exact mass spectrometric strategy for comprehensive and rapid characterization of phenolic compounds in licorice. Rapid Commun. Mass Spectrom. 27, 2297–2309. 10.1002/rcm.6696 24097385

[B52] Yang B. Xuan S. Ruan Q. Jiang S. Cui H. Zhu L. (2020). UPLC/Q-TOF-MS/MS-based metabolomics revealed the lipid-lowering effect of Ilicis Rotundae Cortex on high-fat diet induced hyperlipidemia rats. J. Ethnopharmacol. 256, 112784–112811. 10.1016/j.jep.2020.112784 32222573

[B53] Yang L. Fang Y. Liu R. He J. (2020). Phytochemical analysis, anti-inflammatory, and antioxidant activities of dendropanax dentiger roots. Biomed. Res. Int. 25, 1–13. 10.1155/2020/5084057 PMC7700040 33294445

[B54] Yesmin S. Paul A. Naz T. Rahman A. Akhter S. F. Wahed M. I. I. (2020). Membrane stabilization as a mechanism of the anti-inflammatory activity of ethanolic root extract of Choi (Piper chaba). Clin. Phytoscience 6, 59–10. 10.1186/s40816-020-00207-7

[B55] Zayede D. Mulaw T. Kahaliw W. (2020). Antidiarrheal activity of hydromethanolic root extract and solvent fractions of clutia abyssinica jaub. & spach (Euphorbiaceae) in mice. Evidence-Based Complement. Altern. Med. 2020, 5416749. 10.1155/2020/5416749 PMC7201494 32419812

[B56] Zengin G. Mahomoodally M. F. Aktumsek A. Ceylan R. Uysal S. Mocan A. (2018). Functional constituents of six wild edible silene species: A focus on their phytochemical profiles and bioactive properties. Food Biosci. 23, 75–82. 10.1016/j.fbio.2018.03.010

[B57] Zhuang B. Bi Z. M. Wang Z. Y. Duan L. Liu E. H. (2018). Chemical profiling and quantitation of bioactive compounds in Platycladi Cacumen by UPLC-Q-TOF-MS/MS and UPLC-DAD. J. Pharm. Biomed. Anal. 154, 207–215. 10.1016/j.jpba.2018.03.005 29550710

